# Cellular Heterogeneity and Developmental Dynamics of Aril in Papaya

**DOI:** 10.3390/ijms27093957

**Published:** 2026-04-29

**Authors:** Jin Shi, Yuxin Wang, Ruirong Hu, Yujie Fang, Wen Wang, Ray Ming

**Affiliations:** Center for Genomics and Biotechnology, Fujian Agriculture and Forestry University, Fuzhou 350002, China52462043073@fafu.edu.cn (R.H.);

**Keywords:** *Carica papaya* L., aril, transcriptome, single-cell transcriptome

## Abstract

The papaya aril is a specialized seed appendage that has been reported to contain germination-inhibiting substances and usually requires removal before seed germination, thereby limiting breeding efficiency. However, the cellular origin and candidate molecular regulators of papaya aril development remain poorly understood. To investigate the early developmental process and candidate regulatory genes of the papaya aril, we combined histological analysis, bulk RNA-seq, and single-cell RNA-seq. Histological observations suggested that aril differentiation begins around 10 days after pollination (DAP) in the funiculus region. Based on this initiation stage, bulk RNA-seq profiling of seeds at 5, 10, and 15 DAP identified genes with initiation-stage-specific expression and prioritized candidate genes potentially related to seed appendage development, including *CpRING-like*, *CpMBR2*, and *CpNDR8*. Single-cell RNA-seq of seeds at 10 and 15 DAP annotated a putative aril cell population and reconstructed its developmental trajectory, revealing five trajectory-associated genes: *CpATJ3*, *CpDYL1*, *CpGRP-like*, *CpHIRD11*, and *CpERD15*. Integrative analysis of bulk and single-cell transcriptomic datasets further identified three candidate genes potentially involved in aril development: *CpFER3*, *CpUVI4*, and *CpCEP1*. These findings support the funiculus region as the likely anatomical origin of the papaya aril and provide candidate genes for future functional validation.

## 1. Introduction

Papaya (*Carica papaya* L.) is an economically important fruit crop in tropical and subtropical regions, and its seeds represent the critical germplasm resource for genetic improvement and cultivar development [1]. Mature papaya seeds are black and enveloped by a transparent, membranous aril—a specialized seed appendage widely distributed among both angiosperms and gymnosperms. In many species, such as litchi (*Litchi chinensis*), longan (*Dimocarpus longan*), passion fruit (*Passiflora edulis*), and yew (*Taxus baccata*), the aril is fleshy, brightly colored, and enriched in sugars, lipids, and nutrients. These characteristics attract animals and insects, thereby facilitating seed dispersal [2]. However, in certain species, the arils can suppress germination either through chemical inhibitors or by acting as a physical barrier that restricts water uptake and gas exchange [3,4]. In papaya, the aril also suppresses seed germination and is therefore usually removed before sowing. This requirement substantially increases labor costs and prolongs the breeding cycle. Therefore, clarifying the developmental origin and candidate molecular regulators of the papaya aril may provide a foundation for breeding aril-free cultivars and improving breeding efficiency.

Previous studies have identified several genes and pathways associated with aril development in different species. In litchi, *LcHMG1* and *LcHMG2* regulate aril size by controlling early cell division and late cell expansion, respectively [5]. In *Celastrus orbiculatus*, members of the WRKY, Aux/IAA, ARF, and MADS-box families are involved in aril formation [6], while MADS-box genes regulate aril structural development in ginkgo (*Ginkgo biloba*) and yew (*Taxus* spp.) [7]. In bitter melon (*Momordica charantia*), WRKY transcription factors participate in aril carotenoid accumulation [2]. Additionally, phenylpropanoid biosynthesis, secondary metabolism, and hormone signaling pathways are associated with aril development [8]. Nevertheless, these studies have mainly focused on a limited number of species, and a comprehensive understanding of papaya aril development remains lacking. In particular, the onset of aril cell differentiation and the candidate regulatory genes involved have not yet been systematically elucidated.

Single-cell RNA sequencing (scRNA-seq) enables cell-type-resolved analysis of cellular composition and developmental trajectories during plant organ development [9]. This technology has been widely applied in model plants, including *Arabidopsis thaliana* [10,11], rice (*Oryza sativa*) [12,13], tomato (*Solanum lycopersicum*) [14], and maize (*Zea mays*) [15], as well as in crop species such as tea (*Camellia sinensis*) [16] and cotton (*Gossypium hirsutum*) [17], for cell atlas construction and developmental trajectory inference. To date, however, no study has reported the application of scRNA-seq to papaya aril development. Because papaya aril differentiation occurs in a restricted seed region, scRNA-seq may help resolve developmentally specific cell populations that are masked in bulk-tissue transcriptomic analysis.

In the present study, papaya seed aril development was investigated using an integrated histological and transcriptomic strategy. First, paraffin sectioning was performed to determine the early stage of aril differentiation. Next, stage-specific bulk RNA-seq was used to identify aril-associated differentially expressed genes and infer functional pathways and regulatory networks. In parallel, we constructed a single-cell transcriptomic atlas of papaya seeds to define major cell populations and infer the developmental trajectory of putative aril cells. Finally, candidate genes showing significant changes in both bulk and single-cell transcriptomic analyses were identified through integrative analysis. These results provide candidate cell populations and genes for future functional studies of papaya aril development.

## 2. Results

### 2.1. Morphological Characteristics of Aril Initiation in Papaya

**Figure 1:**
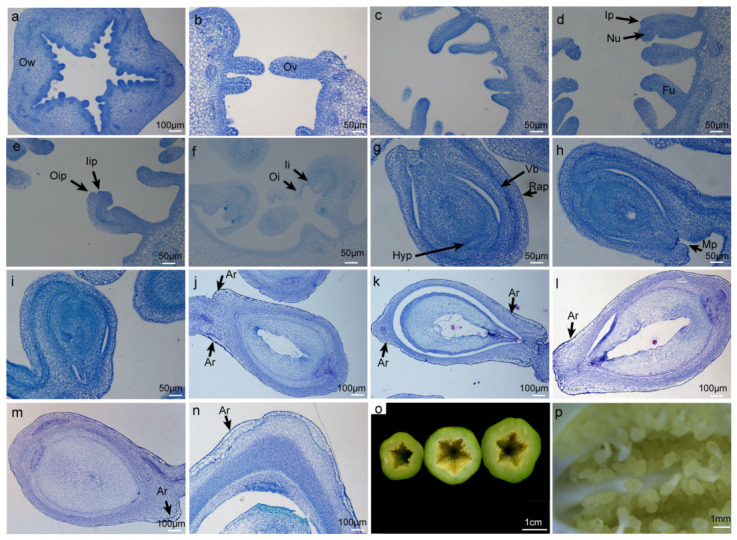
Paraffin sections of papaya ovules and seeds at different developmental stages. (**a**) Cross section of ovary. (**b**–**h**) Paraffin-embedded sections of ovules from flower buds with a diameter of approximately 0.1–2.8 cm. (**i**–**n**) Paraffin sections of seeds after flowering. (**o**) Cross sections of fruits at 5, 10, and 15 days after pollination. (**p**) Stereomicroscopic image of seeds at 5 days after pollination. Ow: Ovary wall, Ov: Ovule primordium, Nu: Nucellus, Ip: Integument primordium, Fu: Funicle, Oip: Outer integument primordium, Iip: Inner integument primordium, Oi: Outer integument, Ii: Inner integument, Vb: Vascular bundle, Rap: Raphe, Mp: Micropyle, Hyp: Hypostase, Ar: Aril. (**a**,**j**–**n**): scale is 100 μm, (**b**–**i**): scale is 50 μm, (**o**): scale is 1 cm, (**p**): scale is 1 mm.

In this study, paraffin sectioning was performed on ovaries and ovules at different developmental stages to observe the developmental processes of the aril and seed structure in papaya. The results showed that papaya ovules are anatropous and possess two integuments (Figure 1a–h). At 5 days after pollination (DAP), no obvious morphological changes were observed compared with ovules at anthesis (2.8 cm). At this stage, the seed structure could be divided into three layers: the outermost layer consisted of outer integument cells and vascular cells; the middle layer comprised inner integument cells, and the innermost layer contained embryo and endosperm cells (Figure 1i). At 10 DAP, a group of cells morphology distinct from surrounding cells appeared near the funiculus end. These cells were larger, rectangular, regularly arranged, and lightly stained and were identified as aril cells (Figure 1j). At 15 DAP, aril cells had extended from the funiculus end toward the chalazal end (Figure 1k). As seed development progressed, the outermost aril cells continued to enlarge and the number of cell layers increased (Figure 1l–n). Collectively, these observations indicate that aril cells first appeared at 10 DAP at the funiculus end.

### 2.2. Transcriptomic Dynamics During Aril Development

**Figure 2:**
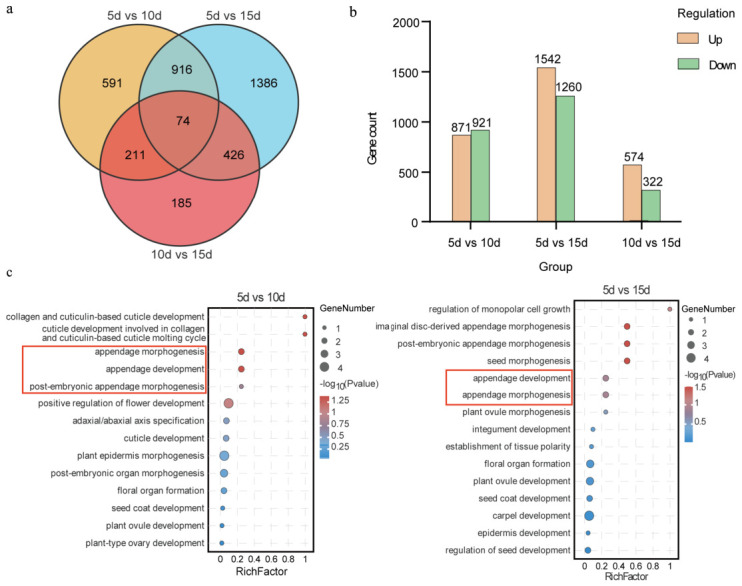
Identification and functional enrichment analysis of differentially expressed genes (DEGs) in papaya seeds at different developmental stages. (**a**) Venn diagram of differentially expressed genes in papaya seeds at different developmental stages. (**b**) Number of up-regulated and down-regulated differentially expressed genes. (**c**) Analysis of up-regulated differential gene GO enrichment in 5 d vs. 10 d and 5 d vs. 15 d. Note: The red lines mark pathways associated with appendage structure development.

To identify genes associated with aril cell development, differential expression analysis was performed using seed transcriptomes at 5, 10, and 15 DAP. Across the three pairwise comparisons, 74 common differentially expressed genes (DEGs) were identified (Figure 2a,b). Because aril initiation and subsequent development occur during these stages, subsequent analyses focused on genes commonly upregulated in “5 d vs. 10 d” and “5 d vs. 15 d”. KEGG enrichment analysis of the upregulated genes showed that the phenylpropanoid biosynthesis pathway was enriched in all three comparisons (Figure S1) and was significantly enriched in both “5 d vs. 10 d” and “5 d vs. 15 d”. In addition, pathways related to plant hormone signal transduction and “stilbenoid, diarylheptanoid and gingerol biosynthesis” were also enriched (Figure S1). 

Further Gene Ontology (GO) enrichment analysis indicated that terms related to the morphology and development of appendage structures were significantly enriched in both “5 d vs. 10 d” and “5 d vs. 15 d” (Figure 2c). Given that the aril is a specialized seed appendage, genes annotated to appendage-development-related GO terms were further examined, including *CpRING-like*, *CpMBR2*, and *CpNDR8* (Table A1). Among them, *CpNDR8* and *CpRING-like* showed continuous upregulation across developmental stages, and *CpRING-like* was also a shared DEG in both “5 d vs. 10d” and “5 d vs. 15 d”. These genes were therefore considered potential candidates associated with early aril formation.

### 2.3. Transcription Factor Profiling and Co-Expression Network Analysis

**Figure 3:**
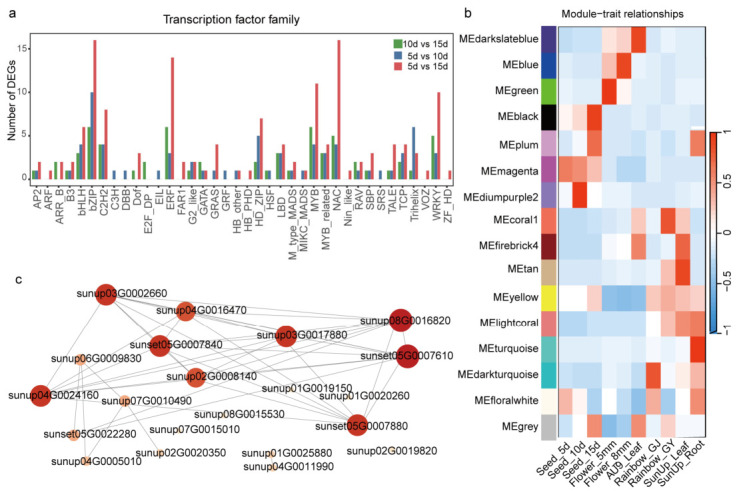
Identification and co-expression network analysis of differentially expressed transcription factors during papaya aril development. (**a**) Identification of different expression transcription factors in papaya arils at different developmental stages. (**b**) Module-organization association analysis. (**c**) Co-expression network of genes associated with plant hormone signaling pathways in the MEblack module. The depth of the color and the size of the dot represent the degree value.

Transcription factor annotation of the upregulated DEGs across the three developmental stages revealed multiple transcription factors potentially involved in aril development. The five largest transcription factor families were bZIP, NAC, ERF, MYB, and WRKY. Among them, bZIP was the most abundant across all comparison groups, whereas NAC transcription factors showed a marked increase in the “5 d vs. 15 d” comparison (Figure 3a). GO enrichment analysis of these transcription factors indicated enrichment in biological processes related to wax biosynthesis as well as flower and seed development, suggesting that these transcription factors may be associated with epidermal/cuticular differentiation during aril formation (Figure S2).

A co-expression network was constructed using Weighted Gene Co-expression Network Analysis (WGCNA), and two modules highly positively correlated with seed tissues were identified: MEmediumpurple2 and MEblack (r > 0.80). Both modules were significantly associated with the 10 DAP and 15 DAP stages (Figure 3b). Functional enrichment analysis showed that MEmediumpurple2 was mainly enriched in DNA replication and RNA-mediated transposition, suggesting enhanced DNA-replication-related activity at 10 DAP, a critical period for seed development (Figure S3). The MEblack module (1420 genes) was significantly enriched in plant hormone signal transduction and galactose metabolism (Figure S4). Plant hormones regulate numerous growth and developmental processes throughout the plant life cycle via complex signaling regulatory networks and play crucial roles in the regulation of cell division, elongation, and differentiation [18].

A gene regulatory network was constructed for genes in this module that are annotated to the plant hormone signal transduction pathway (Figure 3c), and two hub genes were identified: *CpARR11* and *CpABI5* [19,20]. *ARR11* is closely related to cytokinin signaling, whereas *ABI5* functions in abscisic acid (ABA) signaling during seed maturation and germination. These genes were proposed as candidate regulators associated with aril development.

### 2.4. Single-Cell Atlas of Papaya Seeds and Cell Type Identification

**Figure 4:**
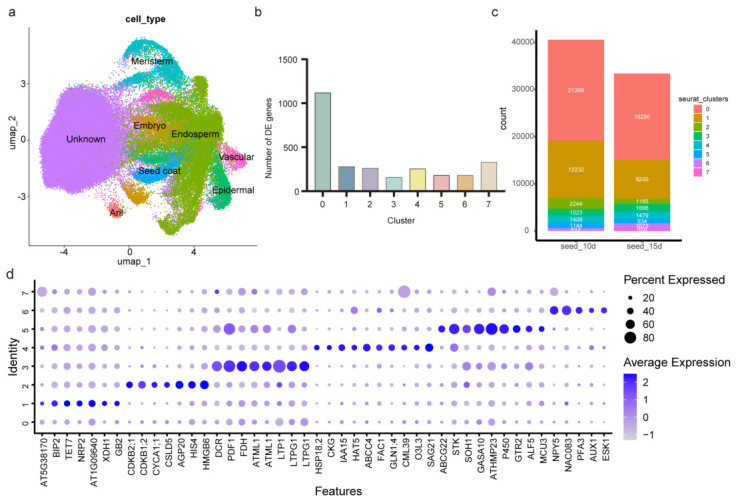
Single-cell transcriptomic characterization of cell populations during papaya seed development. (**a**) UMAP visualization of seven identified cell populations. Note: dots represent single cells, and different colors represent different cell types. (**b**) Number of upregulated differentially expressed genes in each cluster. (**c**) Number of cells in each cluster at two different developmental stages, seed_10d and seed_15d. (**d**) Bubble map of representative subpopulation marker gene expression.

Based on the histological observation that morphologically distinguishable aril cells first appeared near the funiculus at approximately 10 DAP, and considering that 15 DAP represents an early stage of subsequent aril development, seeds at 10 and 15 DAP were selected for single-cell RNA sequencing to capture cellular heterogeneity during aril initiation and early development. A papaya seed single-cell atlas was generated following a standard analysis pipeline (Figure S5). In total, 73,948 high-quality cells and 18,934 genes were retained, and cells were partitioned into eight clusters. The clustering results were visualized using Uniform Manifold Approximation and Projection(UMAP) (Figure 4a). 

Given the lack of systematic single-cell studies and cluster-specific marker genes for papaya seeds, two complementary strategies were used for cell cluster annotation: (i) previously reported plant single-cell marker genes, particularly marker genes identified in *Arabidopsis* seeds, and public single-cell/spatial transcriptomics datasets were used to identify homologous genes in papaya (Figure 4d), and (ii) functional annotation and enrichment analyses were performed for upregulated genes in each cluster (Figure 4c and Figures S6–S11), which were then used to infer and assign cell identities.

In Cluster 1, homologs of *BiP2* and *AT5G38170* were highly expressed. *BiP2* participates in polar nuclei fusion during endosperm nuclear proliferation, and *AT5G38170* is expressed in the endosperm (TAIR) [21]. Moreover, most of the top 20 upregulated genes in this cluster were annotated as endosperm-related genes in *Arabidopsis* seed scRNA-seq data [22]. In Cluster 2, the root meristem marker genes *HMGB6* and *CSLD5* [23], together with multiple cyclin-related genes, were significantly upregulated [24,25]; consistent with this, GO enrichment indicated that upregulated genes were mainly enriched in cell proliferation-related terms (Figure S6). In Cluster 3, seven epidermal marker genes were identified, including *DCR* (DEFECTIVE IN CUTICULAR RIDGES) [23], *PDF1* (PROTODERMAL FACTOR1), and *FDH*. Members of the *LTP* family, *LTPG1*, *LTP1*, and *LTP6*, also showed enriched expression in epidermal cells [26]. In addition, genes in this cluster were enriched in fatty acid modification and wax/cutin biosynthesis pathways (Figures S7 and S8) [27,28]. In Cluster 4, multiple embryo development-related genes were significantly upregulated, and Cluster 4 was therefore annotated as embryo cells [29,30]. In Cluster 5, functional annotation of upregulated genes revealed enrichment of multiple seed coat-related genes [22], and GO terms included lignan biosynthetic processes and glucosinolate transport pathways associated with defense functions (Figure S9). In Cluster 6, homologs of *AUX1* and *ESK1* showed specific or elevated expression. *AUX1* localizes to the apical plasma membrane of protophloem cells, while *ESK1* is highly expressed in xylem cells [23,31]. This cluster also contained cambium-related markers and specific expression of genes involved in xylem differentiation and development [32,33,34,35]. Furthermore, the upregulated genes were enriched in pathways related to phloem/xylem tissue formation and xylem development (Figures S10 and S11), supporting its annotation as vascular tissue cells.

Cluster 7 contained relatively few upregulated genes and lacked clear cluster-specific markers. The dot plot showed that the overall expression levels of the assessed genes were very low in Cluster 7. Due to the current lack of aril cell marker genes, Cluster 7 could not be reliably annotated using existing plant single-cell databases. However, Cluster 7 contained 5 cells at 10 DAP and increased to 559 cells at 15 DAP, consistent with the observed increase in aril cell number between these two stages (Figure 4c). Therefore, Cluster 7 was tentatively assigned as a putative aril-associated cell population, pending further validation.

### 2.5. Reconstruction of the Differentiation Trajectory of Putative Aril Cells

**Figure 5:**
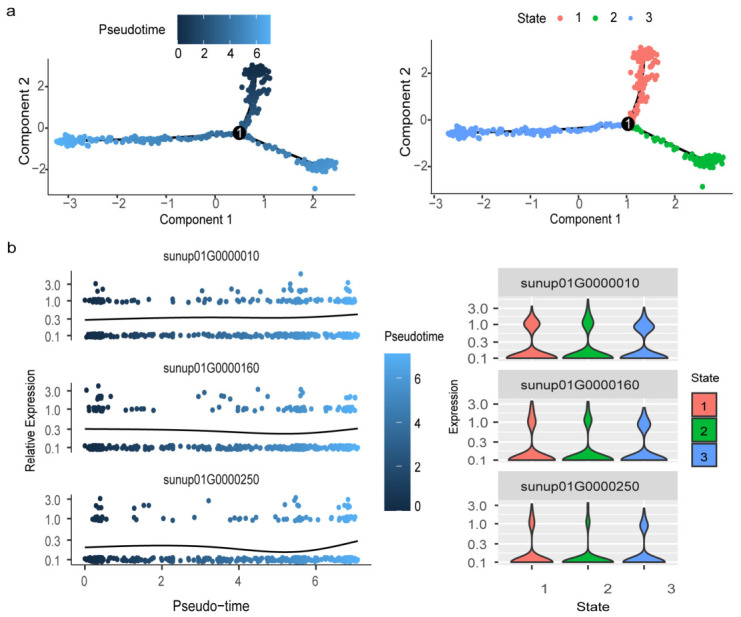
Pseudotime trajectory analysis reveals continuous differentiation of papaya arils. (**a**) Continuous differentiation of arils on the pseudo-timeline.(**b**) Top three genes with the highest contribution to aril development along the pseudotime trajectory. Note: The number “1” in panel (**a**) indicates the root node (starting point) of the pseudotime trajectory, and the black curves in panel (**b**) represent the smoothed fitted trends of gene expression along pseudotime.

To investigate the developmental trajectory of the putative aril cells, pseudotime analysis was performed on cells from Cluster 7 (Figure 5a). The top three genes with the highest contributions to this trajectory were *CpEVI5-like*, *CpGOLGA*, and *CpZCCHC8A* (Figure 5b). Along the pseudotime trajectory, 53 pseudotime-associated genes were identified and grouped into three gene clusters based on their expression patterns; GO enrichment analysis was subsequently performed for each gene set (Figure S12a). Genes in expression cluster 3 were mainly enriched in terms related to the “mitotic cell cycle”, suggesting that cell-cycle-related processes may contribute to early aril cell development.

The BEAM function further identified five genes significantly associated with the developmental trajectory (*q* < 1 × 10^−4^): *CpATJ3*, *CpDYL1*, *CpGRP-like*, *CpHIRD11*, and *CpERD15*. Their expression dynamics along pseudotime were visualized as a heatmap and grouped into four clusters (cluster 1, cluster 2, cluster 3, and cluster 4) (Figure S12b). In *Arabidopsis*, *ATJ3* is involved in plant responses to ABA and affects seed germination and seedling growth [36], and the *Arabidopsis* gene *HIRD11* is closely related to plant growth [37]. These genes were therefore considered trajectory-associated candidates potentially involved in putative aril cell development.

### 2.6. Integrative Transcriptomic Identification of Candidate Genes Associated with Aril Development

Bulk transcriptomic differential expression analysis captures global gene expression changes between developmental stages, whereas single-cell differential expression analysis enables more precise identification of expression changes within specific cell subpopulations. Since histological observations indicated that papaya aril initiation occurs around 10 DAP and early aril development becomes evident by 15 DAP, the 10–15 DAP interval was considered a key transition window for aril development. Therefore, to prioritize candidate genes potentially associated with aril development, DEGs from the bulk transcriptome comparison “10 d vs. 15 d” (896 genes) were integrated with DEGs identified between seed_10d and seed_15d in putative aril cells (Cluster 7; 256 genes). The intersection of these two DEG sets contained 29 shared genes (Figure S13a), which may represent candidate regulators associated with the developmental transition of papaya arils.

Among these 29 shared genes, 19 were upregulated and 10 were downregulated (Figure S13b). GO enrichment analysis of the shared genes revealed enrichment of multiple development-related terms; within these enriched developmental categories, three genes were included: *CpFER3, CpUVI4*, and *CpCEP1* (Figure S13c). In the bulk transcriptome results, *CpFER3* and *CpCEP1* were upregulated, whereas *CpUVI4* was downregulated. These genes may be associated with aril development. For example, *FER3* may participate in iron transport and storage, which is important for cellular metabolism and development; *UVI4* may be related to plant UV responses, and its downregulation may influence stress resistance during aril development; and *CEP1* may be involved in cell wall synthesis or modification, which is relevant to aril cell structure and function [38,39,40].

## 3. Discussion

Previous studies on arils have mainly focused on morphological development, anatomical origin, and bioactive compounds, whereas the genes and regulatory networks underlying aril cell formation remain poorly understood. In this study, we first determined the timing of aril initiation in papaya using paraffin sectioning. On this basis, we integrated bulk transcriptome and single-cell transcriptome data across developmental stages to identify candidate genes potentially involved in regulating aril development.

Based on RNA-seq data from papaya seeds at different developmental stages, we identified DEGs associated with aril development. Notably, genes upregulated in the three developmental comparisons were consistently enriched in phenylpropanoid biosynthesis, plant hormone signal transduction, and “stilbenoid, diarylheptanoid and gingerol biosynthesis” pathways. Further GO enrichment analysis revealed DEGs involved in appendage morphology and development, including *CpRING-like*, *CpMBR2*, and *CpNDR8*. Previous studies have suggested that the aril is a specialized seed appendage in seed plants as an adaptive trait. WGCNA-based co-expression network analysis further identified *CpARR11* and *CpABI5* as candidate genes closely associated with aril development. Functional studies of their Arabidopsis homologs have shown that *ARR11* promotes root cell differentiation by regulating cytokinin signaling [19], whereas *ABI5* mediates abscisic acid (ABA) signaling and regulates seed germination and seedling development [20]. Together, these results suggest that aril development may be closely linked to coordinated regulation by hormone signaling and development-related gene networks.

In this study, we constructed a single-cell transcriptomic atlas of papaya seeds and reconstructed the developmental trajectory of putative aril cells. We identified the top three genes contributing most to the trajectory, *CpEVI5-like, CpGOLGA*, and *CpZCCHC8A*. Based on the reported functions of their Arabidopsis homologs, *CpEVI5-like* may be related to cell-cycle progression, cell division, and membrane trafficking; *CpGOLGA* may be linked to flavonoid biosynthesis; and *CpZCCHC8A* may be involved in plant morphogenesis [23,41]. These genes may participate in regulating aril cell division, flavonoid synthesis, and morphogenesis. Consistently, genes enriched at the early stage of the putative aril trajectory were mainly associated with “mitosis and cell cycle”-related pathways. This pattern is consistent with the proposed three-stage model of aril development, comprising initiation (cell proliferation), growth (predominantly cell expansion), and storage-product accumulation (corresponding to the “maturation” stage) [2]. In addition, we identified five genes significantly associated with the developmental trajectory: *CpATJ3*, *CpDYL1, CpGRP-like*, *CpHIRD11*, and *CpERD15*. Previous studies have reported that *ATJ3* participates in ABA responses and affects seed germination and seedling growth, whereas *HIRD11* promotes plant growth [23,37]. These genes therefore represent promising candidates for further functional characterization during aril development.

By integrating the aril single-cell transcriptome with bulk transcriptome data, we identified 29 shared genes, among which *CpFER3*, *CpUVI4*, and *CpCEP1* were closely associated with development-related pathways. *FER3* encodes a ferritin protein and may influence plant growth, development, and stress responses by regulating iron uptake, transport, and storage [38]. *UVI4* encodes a plant-specific protein; in strawberry (*Fragaria vesca*), *FvUVI4* restricts increases in cell area and number by suppressing endoreduplication and the mitotic cell cycle, thereby limiting the growth of leaves, petals, and fruits [39]. Accordingly, *CpUVI4* may influence aril cell expansion and division through a similar cell-cycle regulatory mechanism during aril development. *CEP1* can modulate hormonal homeostasis and cell division through crosstalk with cytokinin signaling, thereby affecting plant growth and development [40]. Thus, *CpCEP1* may also participate in aril cell division and differentiation via related regulatory mechanisms. Collectively, these findings suggest that papaya aril formation may involve coordinated regulation of nutrient transport, cell-cycle progression, and hormonal homeostasis.

Although this study provides a single-cell transcriptomic framework for exploring papaya aril development, several limitations should be acknowledged. First, for scRNA-seq library construction, seeds from multiple fruits at the same developmental stage were pooled to obtain sufficient viable cells; however, independent biological replicates were not included for each scRNA-seq time point. Second, although scRNA-seq identified a putative aril cell population and candidate genes associated with aril development, spatial validation by RNA FISH or RNA in situ hybridization was not performed in this study. Therefore, the annotation of this cell population, the inferred developmental trajectory, and the functions of the candidate genes remain putative and should be further validated in future studies using replicated scRNA-seq libraries, RNA FISH/RNA in situ hybridization, spatial transcriptomics, and functional genetic approaches.

## 4. Materials and Methods

### 4.1. Plant Materials

Plant materials were collected from female papaya plants of the AU9 cultivar grown at the Yongchun base in Quanzhou, Fujian Province, China. Female flower buds (0.1–2.8 cm in diameter) and fruits at 5, 10, 15, 20, 25, and 30 DAP were sampled. Tissues were cut into appropriate sizes, fixed in an FAA solution (Coolaber, Beijing, China), and used for paraffin embedding. After determining the timing of aril initiation, samples at 5, 10, and 15 DAP were selected for bulk RNA-seq (three biological replicates per stage). In addition, samples at 10 and 15 DAP were used for single-cell transcriptome sequencing. For each scRNA-seq library, seeds collected from multiple fruits at the same developmental stage were pooled to obtain sufficient viable cells for library construction.

### 4.2. Paraffin Sectioning of Papaya Ovules and Seeds

Ovules and seeds at different developmental stages were collected and fixed overnight in FAA solution (50% ethanol, 5% formaldehyde, 5% glacial acetic acid, *v*/*v*/*v*). Samples were dehydrated through a graded ethanol series (Xilong Scientific Co., Ltd., Shantou, Guangdong, China), cleared in a xylene-ethanol series (Xilong Scientific), infiltrated with paraffin (Leica Biosystems, Nussloch, Baden-Württemberg, Germany), and embedded using a graded infiltration procedure. Sections were prepared using a Leica sliding microtome (Leica Biosystems), deparaffinized in xylene, rehydrated through an ethanol series, stained with toluidine blue (Coolaber), rinsed with distilled water, mounted with coverslips, and observed under a light microscope.

### 4.3. Bulk RNA-Seq Processing and Basic Analyses

Raw reads were evaluated using FastQC and trimmed using Trimmomatic (v.0.38) [42]. Clean reads were aligned to the papaya ‘SunUp’ reference genome using HISAT2 (v. 2.2.1) [43]. Transcript abundance was quantified using RSEM. Differentially expressed genes (DEGs) were identified using DESeq2 (v. 1.42.1) [44] with the following thresholds: |log2(fold change)| ≥ 1 and adjusted *p*-value (FDR) ≤ 0.05. Functional annotation was performed against the NCBI non-redundant protein (Nr) database (http://www.ncbi.nlm.nih.gov/RefSeq/) and using eggNOG-mapper (http://eggnog-mapper.embl.de (accessed on 24 April 2026)) [45]. Transcription factor prediction was conducted using PlantTFDB (https://planttfdb.gao-lab.org/prediction.php (accessed on 24 April 2026)). GO and KEGG enrichment analyses were conducted using OmicShare (https://www.omicshare.com/tools/ (accessed on 24 April 2026)).

### 4.4. Weighted Gene Co-Expression Network Analysis (WGCNA)

Weighted gene co-expression network analysis was performed using the WGCNA package in R (v. 4.3.2) [46]. The input expression matrix was normalized using the TMM method implemented in edgeR prior to network construction. Modules were detected and correlated with developmental stages to identify aril-associated co-expression patterns. The parameters were set as follows: network type, unsigned; linkage method, average; module detection, Dynamic Hybrid Tree Cut algorithm; soft-thresholding power, power = 23; tree cutting, deepSplit = 2; minimum module size, minModuleSize = 30; module merging threshold, MEDissThres = 0.6. In total, 16 tissue-associated modules were obtained, and Two modules highly correlated with developmental stages corresponding to aril initiation were selected based on correlation coefficients (r > 0.80). GO and KEGG enrichment analyses of genes within these modules were performed as described above. Module networks were visualized using Cytoscape (v.3.10.3) [47].

### 4.5. Single-Cell Library Construction and Quality Control

Nuclei suspensions were prepared using density-gradient centrifugation. Cell/nuclei counts and viability were assessed using trypan blue staining and a Countess^®^ II Automated Cell Counter (Thermo Fisher Scientific, Waltham, MA, USA). Samples with viability ≥ 90% were retained, and nuclei concentrations were adjusted to ≥1000 nuclei/μL. Single-cell gel bead-in-emulsion (GEM) generation and library construction were performed using the 10× Genomics Chromium platform. Paired-end sequencing was conducted on an Illumina NovaSeq X Plus platform by GeneDenovo Biotechnology Co., Ltd (Guangzhou, China).

Single-cell FASTQ data were processed using Cell Ranger (v.9.0.1) for alignment to the *Carica papaya* reference genome and generation of count matrices and QC metrics. Downstream filtering was performed in Seurat. Cells were retained if they met the following criteria: detected genes per cell, 800–8000. Genes expressed in fewer than three cells were removed.

### 4.6. Dimensionality Reduction, Batch Correction, and Clustering

All single-cell analyses were conducted in Seurat (v.5.1.0) [48]. Expression matrices were normalized using NormalizeData. Highly variable genes (HVGs) were identified using FindVariableFeatures (method = “vst”, nfeatures = 3000), followed by scaling using ScaleData with default settings. Batch effects between the two samples were corrected using Seurat’s integration workflow. Principal component analysis was performed using RunPCA (dims = 50). Clustering was performed using FindClusters (resolution = 0.1). UMAP embeddings were generated for visualization using RunUMAP. For cell-type annotation, reported seed tissue marker genes were collected from PCMDB (Plant Cell Marker DataBase) (https://www.tobaccodb.org/pcmdb/homePage (accessed on 24 April 2026)) [49] and PlantscRNAdb (http://ibi.zju.edu.cn/plantscrnadb/ (accessed on 24 April 2026)) [50], and homologs in papaya were used to support cluster annotation.

### 4.7. Differential Expression in Cell Subpopulations and Trajectory Inference

Marker genes for each cluster were identified using the Wilcoxon rank-sum test implemented in Seurat (v.5.1.0) [51]. Genes were considered significantly upregulated in a given cluster when they met all of the following criteria: avg_log2FC ≥ 0.25, *p* ≤ 0.05, and expression in ≥25% of cells in the target cluster.

Trajectory inference was performed using Monocle2 (v.2.30.0) [52]. Putative aril cells were ordered along pseudotime to reconstruct developmental progression. Dimensionality reduction was performed using DDRTree with reduceDimension (max_components = 2, method = “DDRTree”), followed by cell ordering to generate trajectories. Differentially expressed genes along pseudotime were identified using Monocle2. Branch expression analysis modeling (BEAM) [53] was applied to detect branch-dependent DEGs after specified branch points, and branch-associated genes were visualized.

## 5. Conclusions

In this study, we systematically characterized the histomorphological changes in papaya (*Carica papaya* L.) ovules and seeds across developmental stages using paraffin sectioning, thereby defining the temporal onset of aril initiation. By integrating bulk RNA sequencing and single-cell transcriptomic analyses, we identified developmental stage-associated transcriptional changes, reconstructed the developmental trajectory of putative aril cells, and prioritized candidate genes potentially involved in aril formation. Although further functional validation is required, this study provides a cellular and molecular framework for investigating papaya aril development and offers candidate targets for future studies aimed at understanding and manipulating aril formation in papaya.

## Data Availability

The raw transcriptome sequencing data of papaya (*Carica papaya* L.) have been deposited in the National Genomics Data Center, Beijing Institute of Genomics (BIG), Chinese Academy of Sciences and China National Center for Bioinformation, under accession numbers CRA040362 (single-cell transcriptome) and CRA040081 (transcriptome). All data relevant to this study are included in the Supplementary Materials accompanying this article.

## Supplementary Materials

The following supporting information can be downloaded at: https://www.mdpi.com/article/10.3390/ijms27093957/s1.

## Appendix A

**Table A1:** Nomenclature and locus IDs of selected genes in *Carica papaya* (SunUp genome).

Gene Name (This Study)	Genome Locus ID
*CpRING-like*	*sunup02G0005780*
*CpMBR2*	*sunup06G0001740*
*CpNDR8*	*sunup01G0020230*
*CpARR11*	*sunset05G0007610*
*CpABI5*	*sunup08G0016820*
*CpEVI5-like*	*sunup01G0000010*
*CpGOLGA*	*sunup01G0000160*
*CpZCCHC8A*	*sunup01G0000250*
*CpATJ3*	*sunup08G0011250*
*CpDYL1*	*sunup07G0004650*
*CpGRP-like*	*sunup03G0011990*
*CpHIRD11*	*sunup06G0020900*
*CpERD15*	*sunup02G0012490*
*CpFER3*	*sunup08G0015050*
*CpUVI4*	*sunup08G0019440*
*CpCEP1*	*sunup09G0011900*
