# Supplementary material for: Cellular Heterogeneity and Developmental Dynamics of Aril in Papaya
Source: Int J Mol Sci. 2026 Apr 29;27(9):3957. doi: 10.3390/ijms27093957 (PMC13163318; doi:10.3390/ijms27093957)
Supplement: Supplementary file 1 [file ijms-27-03957-s001.zip › supplementary figures.pdf]

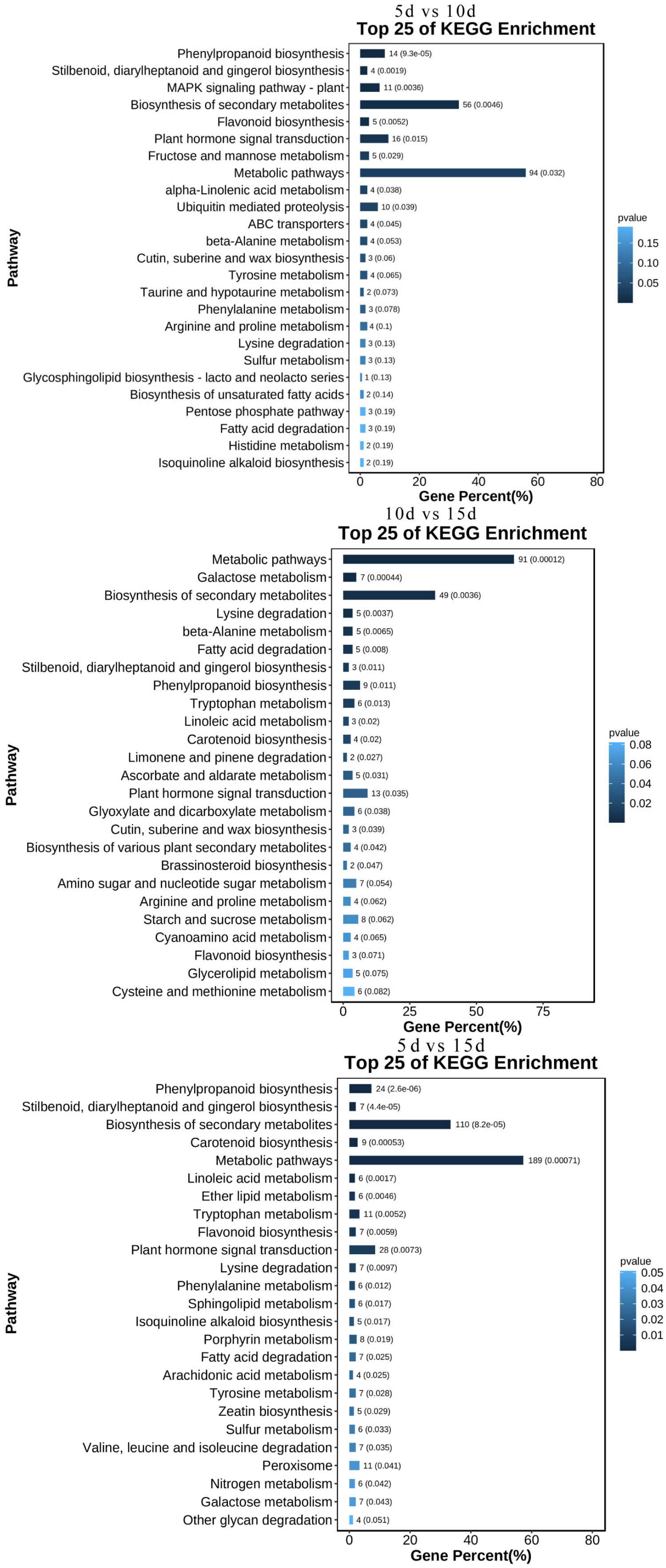

Figure S1. KEGG enrichment analysis of upregulated differential genes in the three comparison groups.

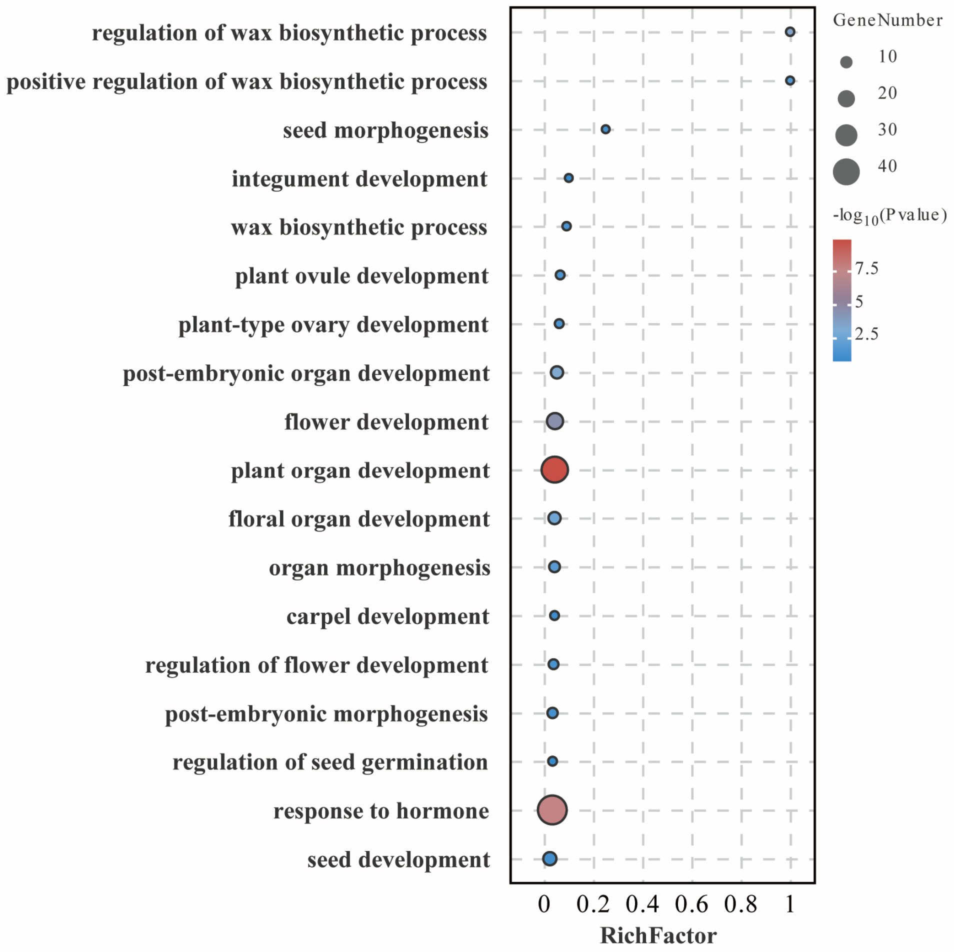

Figure S2. Enrichment analysis of different-expressed transcription factor GO in papaya arils at different developmental stages.

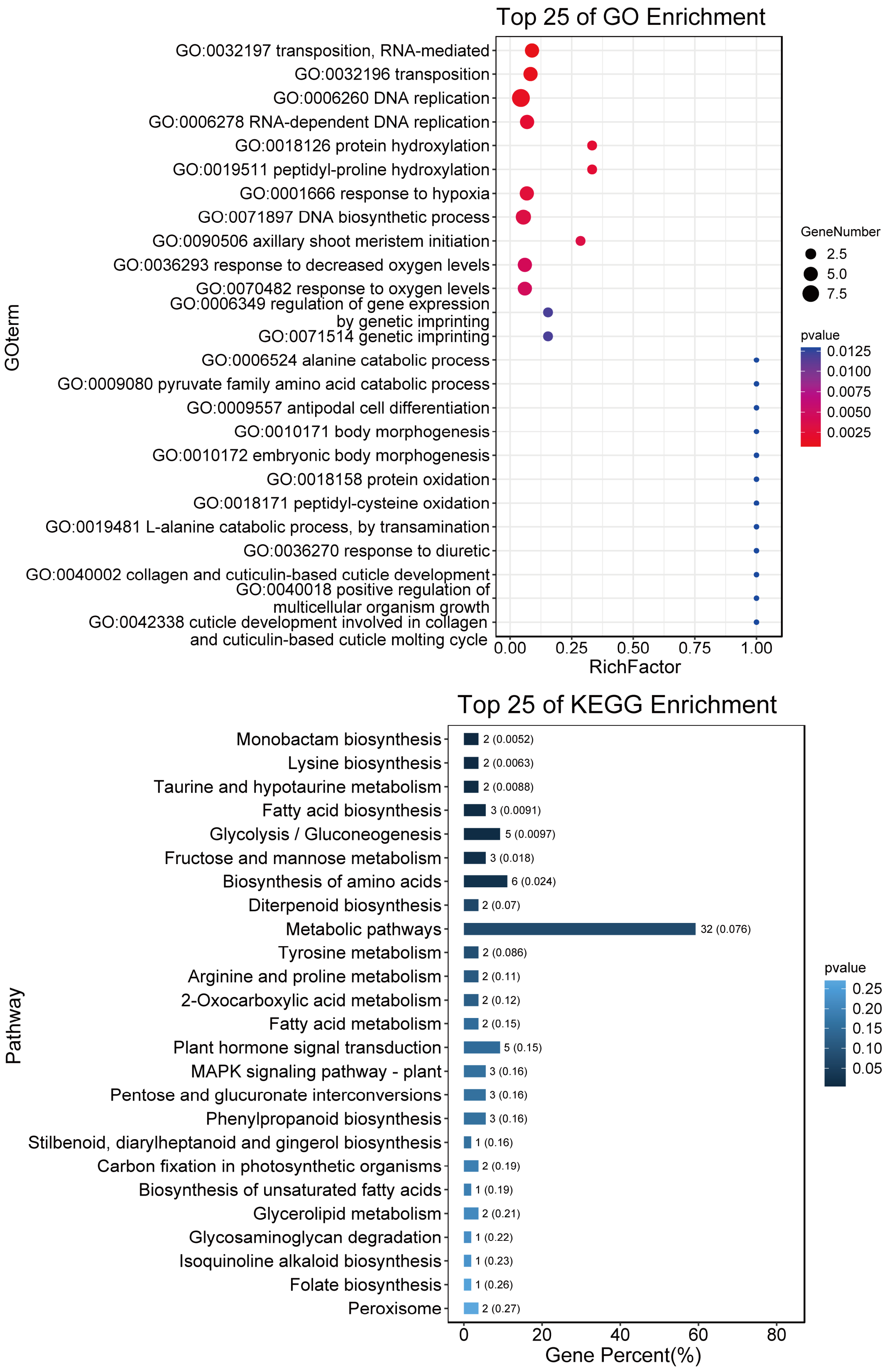

Figure S3. GO and KEGG enrichment analysis of genes in MEmediumpurple2 modules.

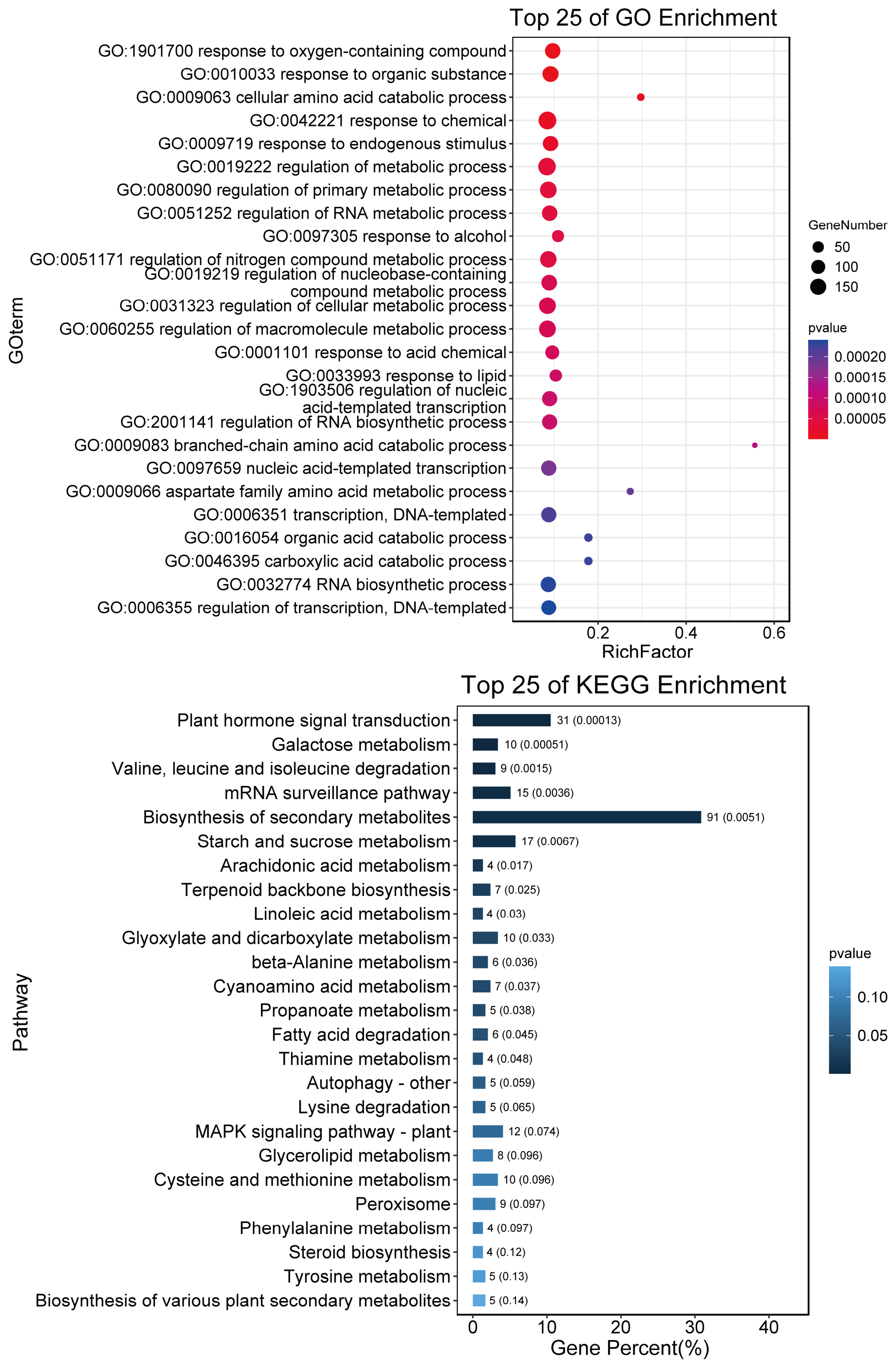

Figure S4. GO and KEGG enrichment analysis of genes in Meblack modules.

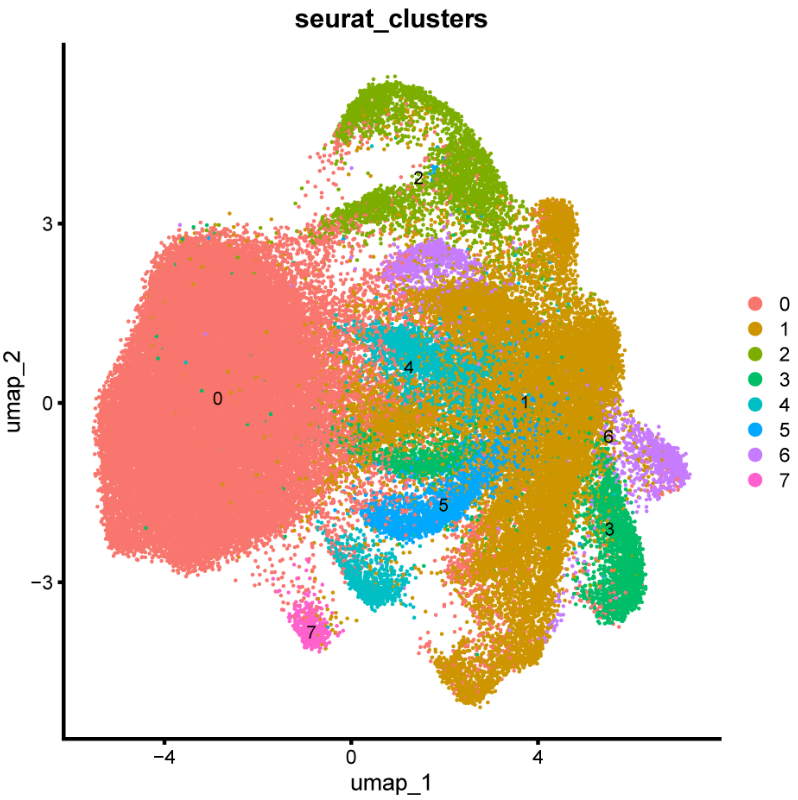

Figure S5. UMAP visualization of 8 cell types of seeds. Note: dots represent single cells, and different colors represent different cell clusters.

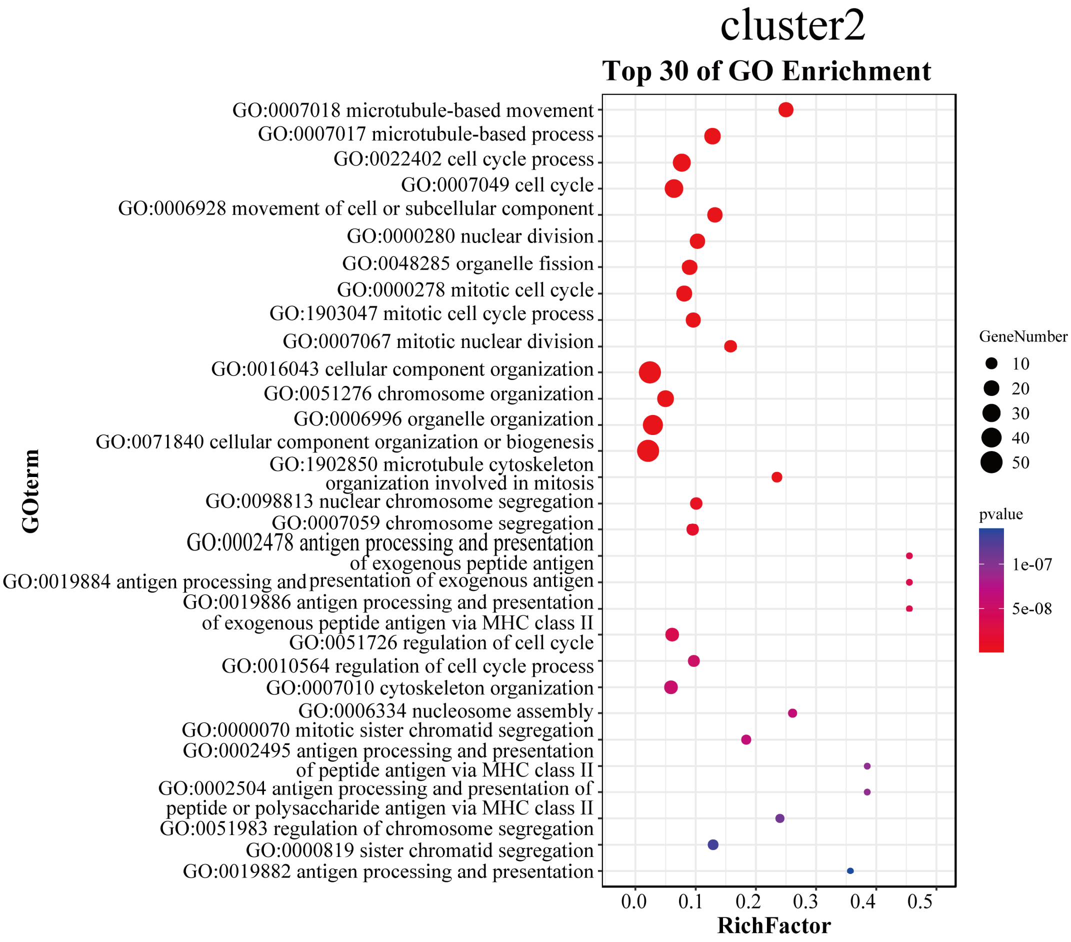

Figure S6. GO enrichment analysis of upregulated genes in Cluster 2.

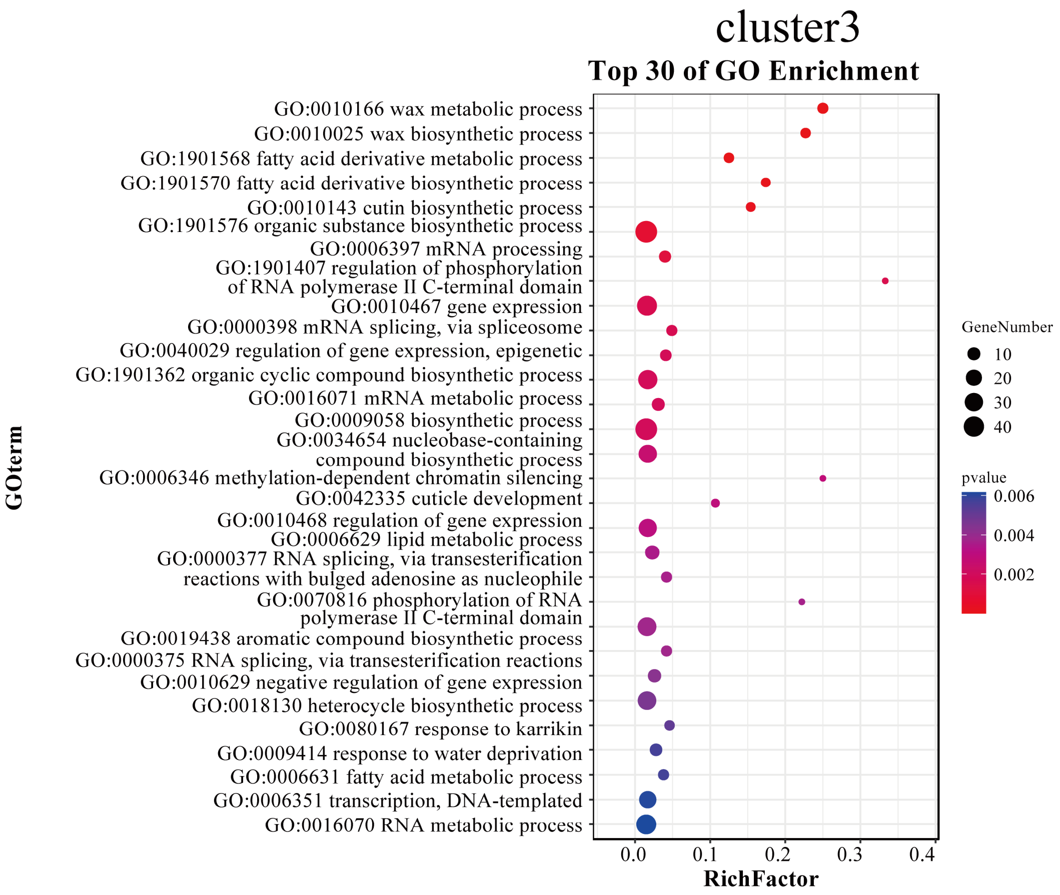

Figure S7. GO enrichment analysis of upregulated genes in Cluster 3.

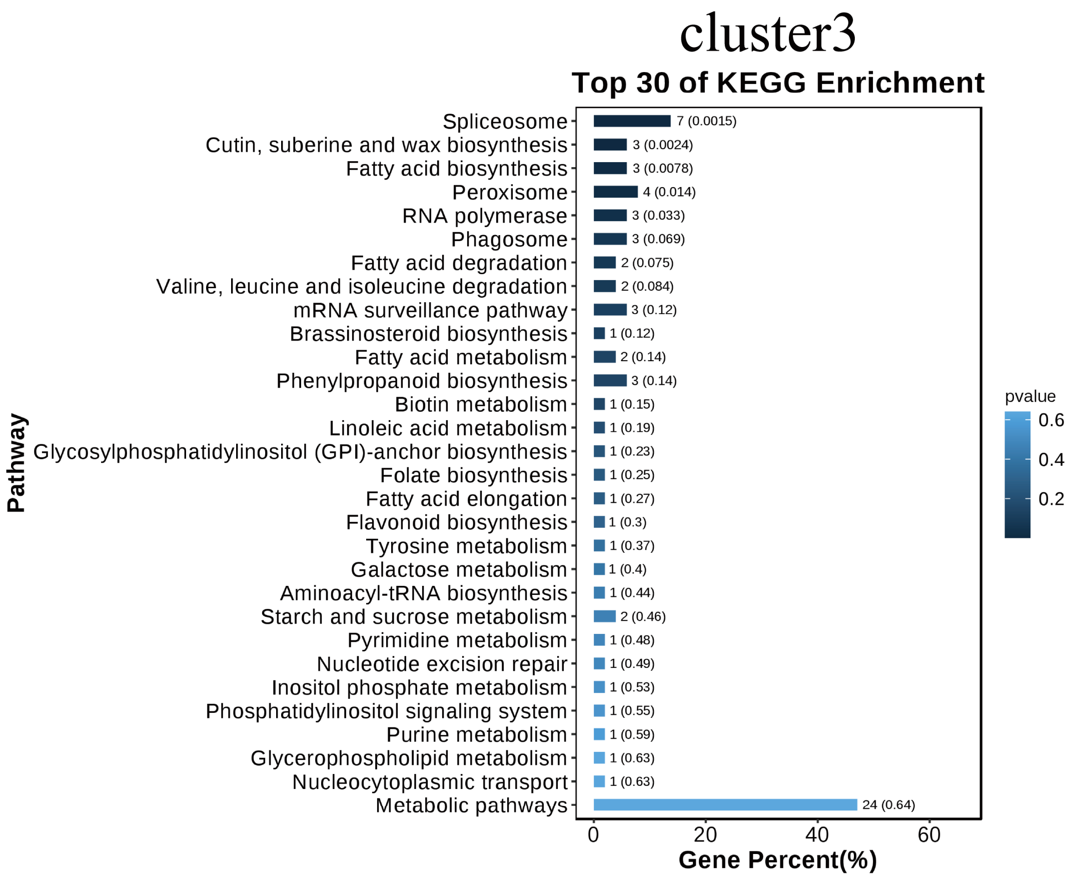

Figure S8. KEGG enrichment analysis of upregulated genes in Cluster 3.

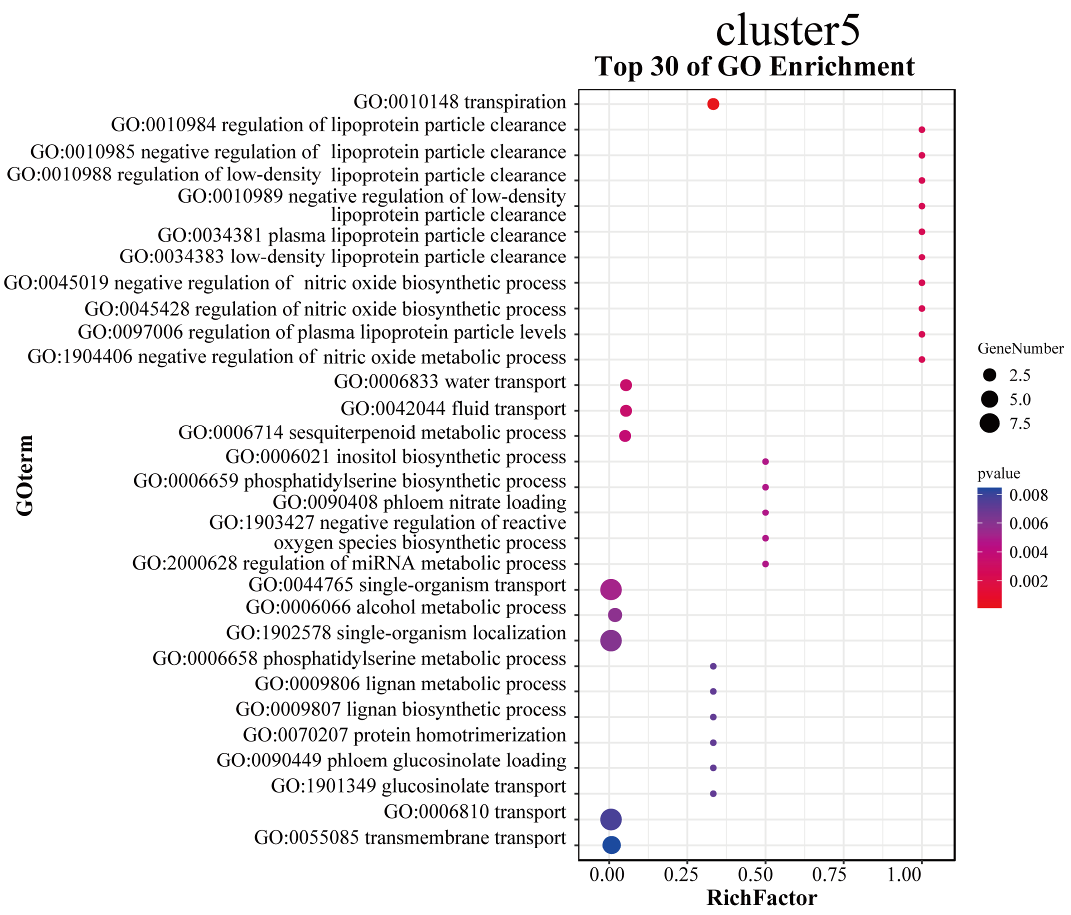

Figure S9. GO enrichment analysis of upregulated genes in Cluster 5.

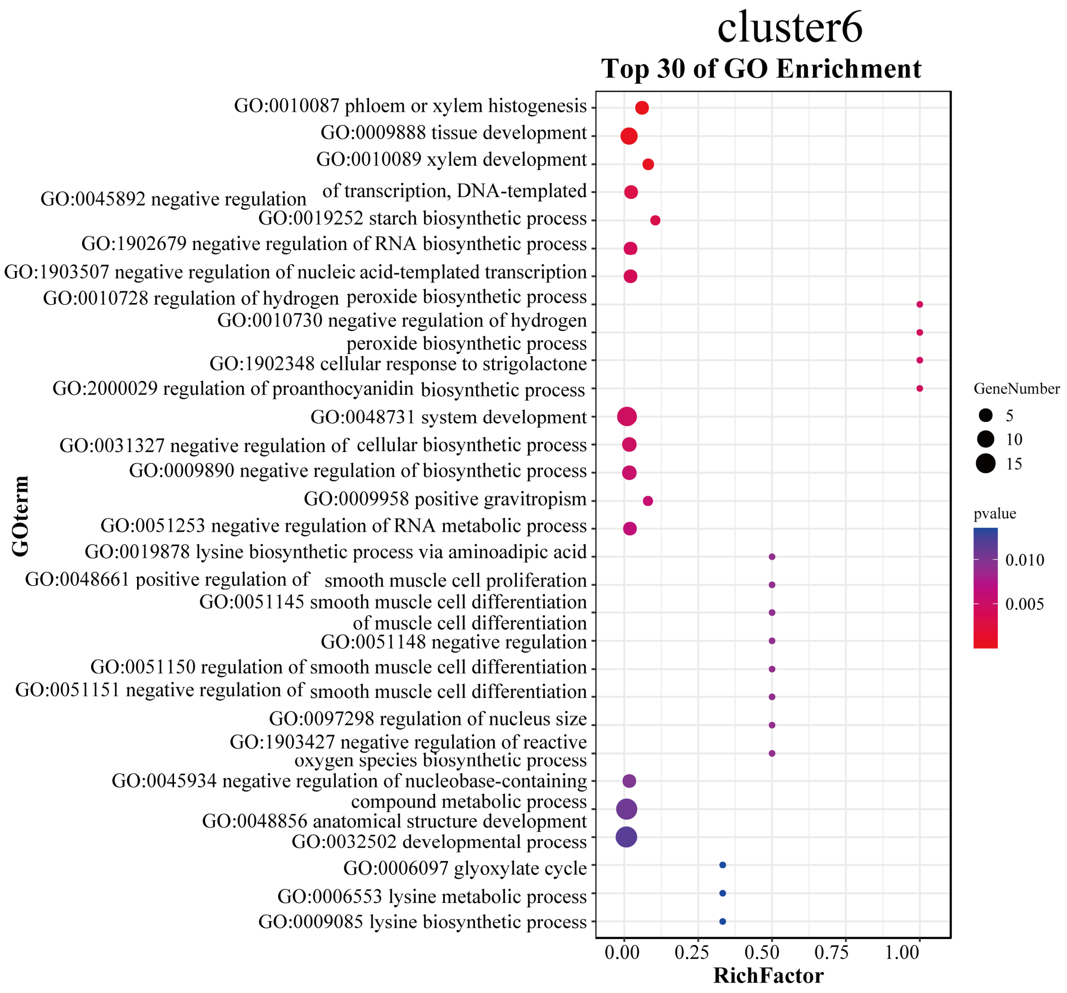

Figure S10. GO enrichment analysis of upregulated genes in Cluster 6.

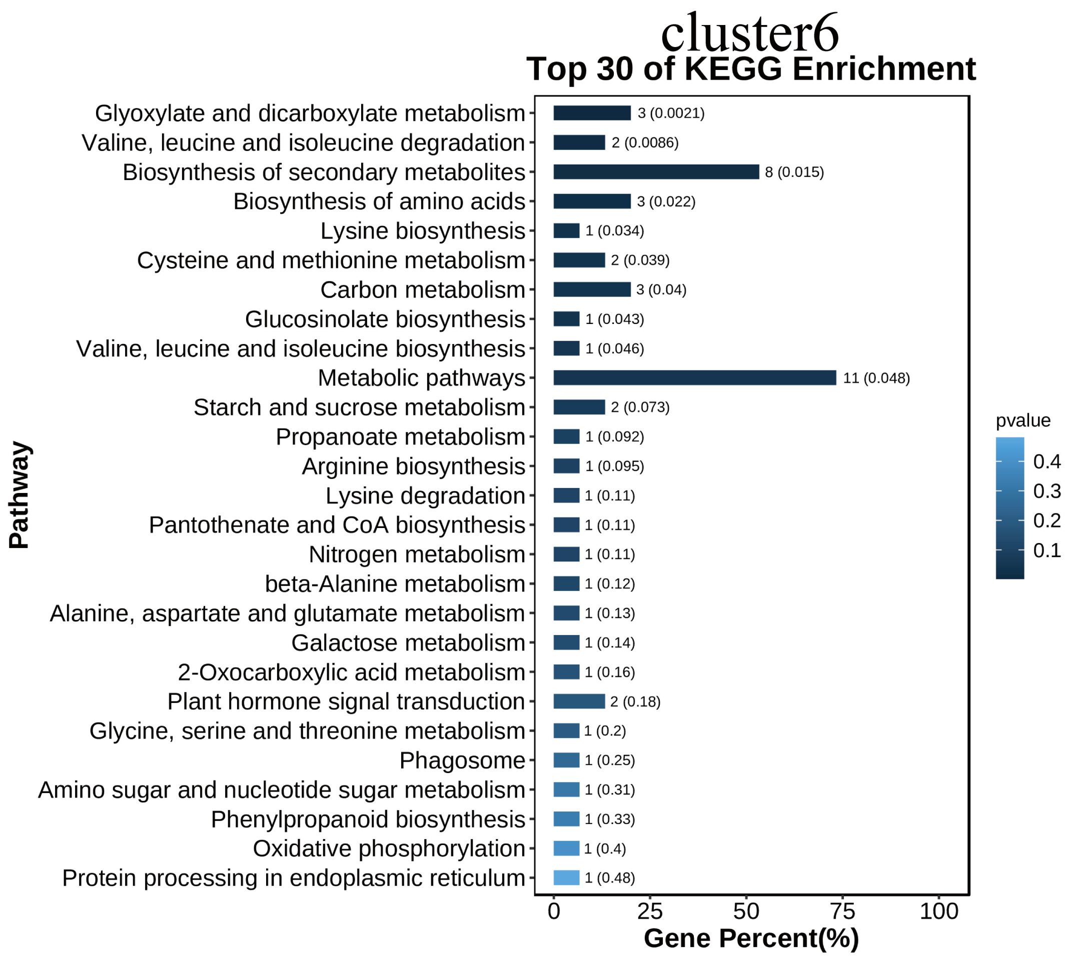

Figure S11. KEGG enrichment analysis of upregulated genes in Cluster 6.

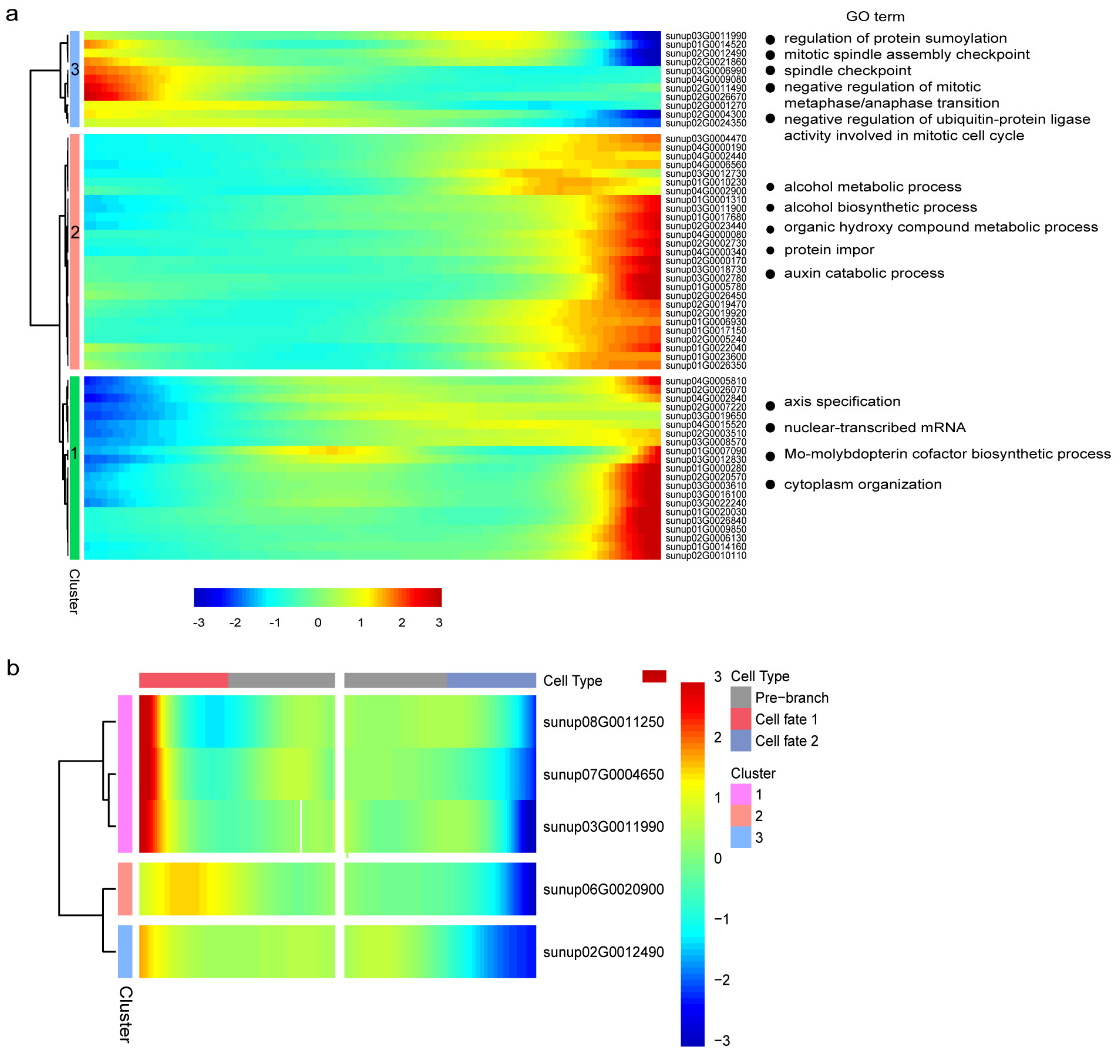

Figure S12. Pseudotime-dependent gene expression dynamics during papaya aril development. (a) Pseudo-timeline-dependent gene expression heat map. (b) Branch-dependent gene expression heat map.

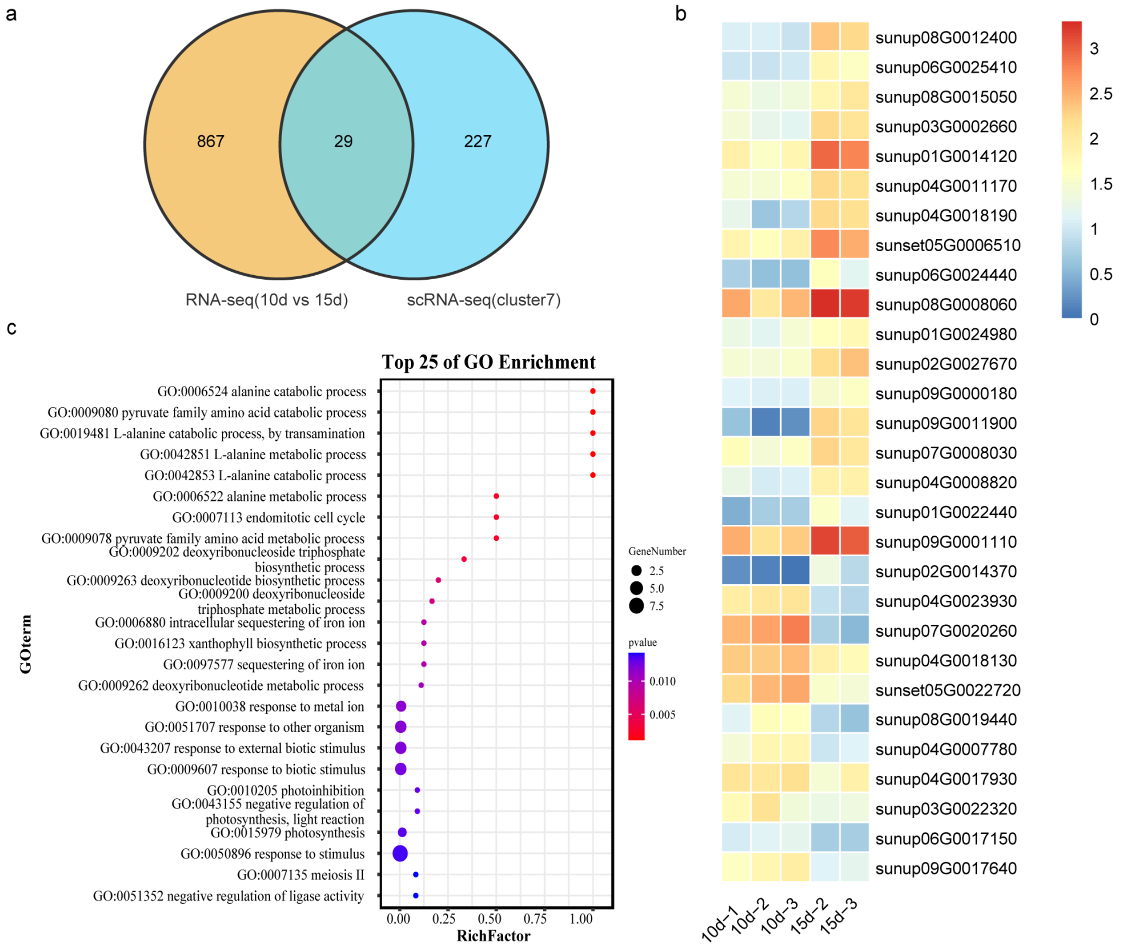

Figure S13. The number of different genes shared between the transcriptome and the single-cell transcriptome. (a) The expression of 29 common genes in the transcriptome. (b) GO enrichment analysis of 29 common genes. (c)
